# Consequences of the historical demography on the global population structure of two highly migratory cosmopolitan marine fishes: the yellowfin tuna (*Thunnus albacares*) and the skipjack tuna (*Katsuwonus pelamis*)

**DOI:** 10.1186/1471-2148-5-19

**Published:** 2005-02-22

**Authors:** Bert Ely, Jordi Viñas, Jaime R Alvarado Bremer, Donna Black, Luciano Lucas, Kelly Covello, Alexis V Labrie, Eric Thelen

**Affiliations:** 1 Department of Biological Sciences, University of South Carolina, Columbia, SC 29208, USA; 2 Institut de Ciències del Mar, C.S.I.C., Passeig Marítim, 37–49, 08003 Barcelona, Spain; 3 Department of Marine Biology, Texas A&M University at Galveston, Galveston, TX 77551, USA; 4 TAMU, Department of Wildlife and Fisheries Sciences, 210 Nagle Hall, TAMU 2258, College Station, TX 77843, USA

## Abstract

**Background:**

Yellowfin and skipjack tuna are globally distributed in the world's tropical and sub-tropical oceans. Since little, if any, migration of these fishes occurs between the Atlantic and Indo-Pacific Oceans, one might expect to see genetic differences between sub-populations in these ocean basins. However, yellowfin and skipjack tuna have extremely large population sizes. Thus, the rate of genetic drift should be slower than that observed for other tunas.

**Results:**

Low levels of genetic differentiation were observed between Atlantic and Pacific samples of yellowfin tuna. In contrast, no genetic differentiation was observed between Atlantic and Pacific samples of skipjack tuna.

**Conclusion:**

Much lower levels of genetic differentiation were found among sub-populations of yellowfin tuna compared to those observed for other large tunas, probably due to the large population size of yellowfin tuna. Since skipjack tuna appear to have even larger population sizes, it is not surprising that no genetic differentiation was detected between Atlantic and Pacific samples of these fish.

## Background

The yellowfin tuna, *Thunnus albacares*, has a global distribution in tropical and sub-tropical oceans. Annual catches of yellowfin tuna have averaged 1.2 million metric tons since 1998 with sizes ranging from 5 to 20 kg [1]. If the average size of a harvested fish is 10 kg, then the harvest of 1.3 million metric tons in 2002 represents approximately 130 million individual fish.

In an earlier study, Scoles and Graves [2] were unable to find evidence of genetic differentiation between small samples of yellowfin tuna (*Thunnus albacares*) from the Atlantic (n = 20) and Pacific Oceans (n = 100) using an RFLP analysis of whole mitochondrial DNA (mtDNA). Subsequently, Ward et al. [3] found significant differentiation at the GPI-A* allozyme locus but only weak evidence for genetic differentiation with an RFLP analysis of whole mtDNA. In the whole mtDNA study, much larger sample sizes were employed to increase the sensitivity of the mtDNA assay, but only two restriction enzymes were used and consequently, only a few restriction sites were analyzed. The GPI-A* data were consistent with earlier studies that demonstrated genetic differentiation between Eastern and Western Pacific samples [4,5].

Skipjack tuna (*Katsuwonus pelamis*) are found in tropical and warm temperate waters of the world's oceans. They are present in the three major oceans in large numbers and comprise approximately 40% of the annual catch of the world's tunas. Annual catches are on the order of 2 million metric tons or approximately 670 million individuals per year [1]. Despite their huge numbers, the skipjack tuna are not as well studied as most members of the genus *Thunnus*. Tagging studies have demonstrated limited seasonal movements, but not much transoceanic movement. Thus, they probably do not spawn at discrete locations [6].

In the Pacific, genetic studies using isozymes have demonstrated an East-West cline in a serum esterase allele [7-9]. Fujino et al. [10] also demonstrated differences in esterase allele frequencies in samples from the Atlantic, Indian, and Pacific Oceans. However, small samples of Atlantic (n = 7) and Pacific (n = 9) skipjack appeared identical when mtDNA was examined [11].

After the formation of the Isthmus of Panama, the potential contact between Atlantic and Indo-Pacific populations was limited to the waters around southern Africa. It appears that this separation of the Atlantic and Pacific Oceans resulted in significant genetic drift for many large pelagic fishes since these subpopulations are now genetically differentiated. Examples include bigeye tuna [12,13], albacore [14,15], swordfish [16,17], blue marlin [18] and sailfish [19]. In addition, even more pronounced differentiation produced species pairs of Atlantic and Pacific bluefin tunas and the Atlantic white marlin and the Pacific striped marlin [19-22].

To measure the degree of genetic differentiation between the Atlantic and Pacific sub-populations of either yellowfin or skipjack tuna, we examined the hypervariable control region I (CR-I) and a segment of a coding region gene of the respective mitochondrial DNAs. Genetic differences were observed between the Atlantic and Pacific yellowfin tuna samples with PCR-RFLP data of the ATCO gene region, but not with CR-I sequence data. In contrast, no differences between the Atlantic and Pacific skipjack tuna samples were detected with either type of data. Information contained in the CR-I reveals very different demographic histories for yellowfin tuna and skipjack tuna. However, very large long-term female effective population sizes (N_e_) were estimated for both species, which may explain the observed levels of inter-oceanic genetic partitioning.

## Results

### Control region nucleotide sequence analysis

**Figure 1:**
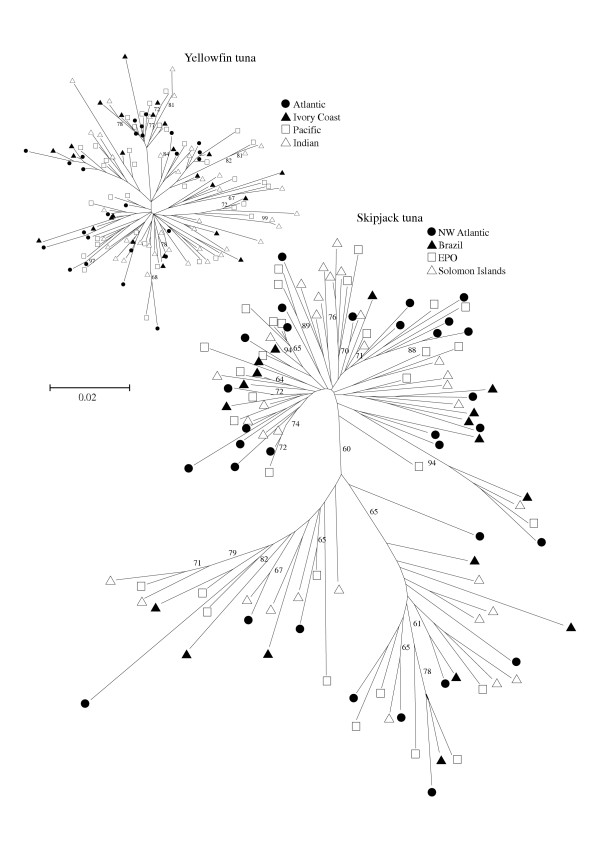
Unrooted neighbor-joining (NJ) trees showing the relationship of 111 yellowfin tuna and 130 skipjack tuna haplotypes estimated from a matrix of Tamura Nei (α = 0.5) distances. Values of bootstrap proportion support above 60% are included and the geographical origin of haplotypes is identified with symbols (see inset). The bar indicates the size of a line representing differences of 2% with both trees drawn to the same scale.

A total of 333 bp of the nucleotide sequence of the mitochondrial DNA (mtDNA) control region was determined for 148 yellowfin tuna (Table 1 and GenBank accession numbers AY899520 – AY899681). For the pooled sample of yellowfin tuna, 110 variable sites defined 130 haplotypes (*h *= 0.997) and a nucleotide diversity (π) of 3.5%. Diversity indices for each locality sampled were also high, although values of π and *h *were slightly lower for the NW Atlantic yellowfin sample where five haplotypes were repeated twice. For skipjack tuna, a total of 394 bp was determined for 115 individuals (Table 2 and GenBank accession numbers AY899405 – AY899519). In the pooled skipjack tuna sample, there were 157 variable sites defining 111 haplotypes, resulting in a very high value of haplotypic diversity (*h *= 0.999). Nucleotide diversity (π = 8.4%) was more than twice as high in skipjack tuna as in yellowfin tuna. All of the sampling localities of skipjack tuna had very high diversity values (π >7.7%; *h *> 0.998). For both species, the high haplotypic diversity values are consistent with the observed large census population sizes (N_c_). Phylogenetic analyses resulted in very different trees for each species. The CR-I gene-tree topology of skipjack tuna is much larger and better structured than the yellowfin tuna phylogeny, and contains multiple branches with high bootstrap proportion support (Figure 1). However, there is no obvious phylogeographic association in either species, with CR-I lineages from different basins scattered throughout the phylogenetic trees. As a consequence, in both yellowfin tuna (Table 3) and skipjack tuna (Table 4), the majority of the genetic variation corresponds to differences between individuals within populations, and only a minor fraction of the variation corresponds to differences among-groups. Thus, in both species, the control region sequences provide no evidence of genetic differentiation between Atlantic and Indo-Pacific sub-populations.

**Table 1:** Genetic diversity indices and demographic parameters of yellowfin tuna CR-I. *N *number of individuals; M, number of haplotypes; *π*, nucleotide diversity; *h*, haplotypic diversity. Tajima's D neutrality test and associated probability in parentheses. Mismatch distribution parameters τ, Θ_O, _Θ_1._

Population	*N*	M	*π*	*h*	Tajima's *D*	τ	Θ_O_	Θ_1_
All	148	130	0.035 ± 0.018	0.997 ± 0.001	-1.588 (0.022)	8.516	0.047	566
NW Atlantic	31	26	0.027 ± 0.014	0.987 ± 0.012	-0.908 (0.185)	9.746	0.000	87
Ivory Coast	32	29	0.029 ± 0.016	0.994 ± 0.009	-1.254 (0.085)	8.531	0.008	1097
Pacific Ocean	41	40	0.033 ± 0.017	0.999 ± 0.006	-1.155 (0.111)	9.250	0.000	6655
Indian Ocean	44	41	0.032 ± 0.017	0.997 ± 0.006	-1.346 (0.059)	8.391	0.708	352

**Table 2:** Genetic diversity indices and demographic parameters of skipjack tuna CR-I. Abbreviations and notations as in Table 1.

Population	*N*	M	*π*	*h*	Tajima's D	τ	Θ_O_	Θ_1_
All	115	111	0.084 ± 0.041	0.999 ± 0.001	-0.419 (0.424)	15.82	12.34	3795

NW Atlantic	31	30	0.082 ± 0.041	0.998 ± 0.009	-0.343 (0.408)	15.32	13.50	6655
Brazil	17	17	0.084 ± 0.043	1.000 ± 0.020	-0.354 (0.431)	28.38	4.03	98
E. Pacific Ocean	32	31	0.077 ± 0.038	0.998 ± 0.008	-0.084 (0.537)	14.00	16.10	6655
Solomon Islands	35	34	0.083 ± 0.041	0.998 ± 0.007	-0.266 (0.445)	14.18	16.75	4683

**Table 3:** AMOVA of the patterns of sequence variation contained in the CR-I of yellowfin tuna. Localities were assigned into three regional groupings: Atlantic, Pacific and Indian. The Atlantic region included two samples, NW Atlantic and Ivory Coast, whereas the Pacific and Indian regions included only one sample each.

Source of Variation	Variance components	Percentage variation	Fixation Indices	Probabilities
Among groups	0.00155 Va	0.31	Φ_CT _: 0.003	0.17 ± 0.01
Among populations within groups	-0.00041 Vb	-0.08	Φ_SC _: -0.001	0.51 ± 0.01
Within Populations	0.49818 Vc	99.77	Φ_ST _: 0.002	0.038 ± 0.006

**Table 4:** AMOVA of the patterns of sequence variation contained in the CR-I of skipjack tuna. Localities were assigned into three regional groupings: Atlantic, Pacific and Indian. The Atlantic region included two samples, NW Atlantic and Brazil, whereas the eastern Pacific and Indian Ocean (Solomon Islands) regions included only one sample each.

Source of Variation	Variance components	Percentage variation	Fixation Indices	Probabilities
Among groups	0.296 Va	1.89	Φ_CT _: 0.019	0.16 ± 0.00
Among populations within groups	0.349 Vb	-2.23	Φ_SC _: -0.023	0.72 ± 0.01
Within Populations	15.712 Vc	100.34	Φ_ST _: -0.003	0.72 ± 0.01

### RFLP analysis of the mitochondrial ATCO gene region

We hypothesized that the high mutation rate of the control region might have resulted in homoplasy and high levels of haplotypic diversity that masked the actual genetic divergence of Atlantic and Pacific mtDNAs in these species. Analysis of a gene that has a lower mutation rate than that of the control region has been used to reveal genetic divergence in bigeye tuna [13]. Therefore, we performed a nucleotide sequence analysis on the yellowfin tuna mitochondrial ATCO gene region since variation had been shown previously for this gene [23,24]. We found mutations at two positions that resulted in the loss of restriction sites for the enzymes *Dde*I and *Hpy*CH4III, respectively. When amplified Atlantic and Indo-Pacific yellowfin ATCO DNAs were analyzed with these two enzymes, most DNAs were cut with both enzymes (Table 5). However, DNAs that had lost either one of the two restriction sites were more common in the Atlantic sample than in the Indo-Pacific sample. When the haplotype distributions were compared, the differences were found to be significant with 7% of the variation occurring between samples (Table 6). Thus, low levels of genetic differentiation have occurred between the two yellowfin sub-populations. When the haplotypes were mapped to a neighbour-joining tree of the control region sequences, the mutations affecting the restriction sites were clustered, suggesting that each mutation had occurred once prior to separation of the two sub-populations (data not shown). This result is consistent with a slower rate of mutation in the ATCO gene region compared to that of the control region.

**Table 5:** Allele frequencies at the yellowfin tuna mtDNA ATPase6 locus

Sample	N	ATCO Haplotypes		
		D	H	DH

Atlantic	138	0.13	0.22	0.65
Pacific	96	0.02	0.12	0.86

Haplotype designations: D, cut with *Dde*I; H, cut with *Hpy*CH4III; DH, cut with both enzymes. N is the sample size.

**Table 6:** Analysis of molecular variance between Atlantic and Pacific yellowfin tuna samples

Source of Variation	d. f.	Sum of Squares	Variance Components	Percentage of Variation
Between Populations	1	1.19	0.015 Va	7.0
Within Populations	232	46.69	0.201 Vb	93.0
Totals	233	48.60	0.216	

Fixation Index F_ST _: 0.070

Va and F_ST _: *P*(random value > = observed value) = 0.00098+/-0.00098

Similar experiments were performed with the skipjack tuna samples. Two variable sites had been observed in the mitochondrial cytochrome b (*cytB*) gene by Terol et al. [25]. Since one of these sites could not be assayed with restriction enzymes, we determined the nucleotide sequence of this portion of the *cytB *gene for each DNA in the Atlantic and Pacific skipjack tuna samples. Little additional variation was observed, and allele frequencies were similar in the two samples (Table 7). When the haplotype distributions were compared, all of the variation occurred within samples. Thus, there is no evidence for genetic differentiation between the Atlantic and Pacific sub-populations of skipjack tuna.

**Table 7:** Allele frequencies at the skipjack mtDNA *cytB *locus

		Allele*		
Region	n	AT	AC	GC

Pacific	80	0.31	0.24	0.45
Atlantic	49	0.31	0.18	0.51

*Variable base at the *Psh*A1 site and a position 19 bases upstream as determined by nucleotide sequence analysis.

### Demographic history and effective population size

**Figure 2:**
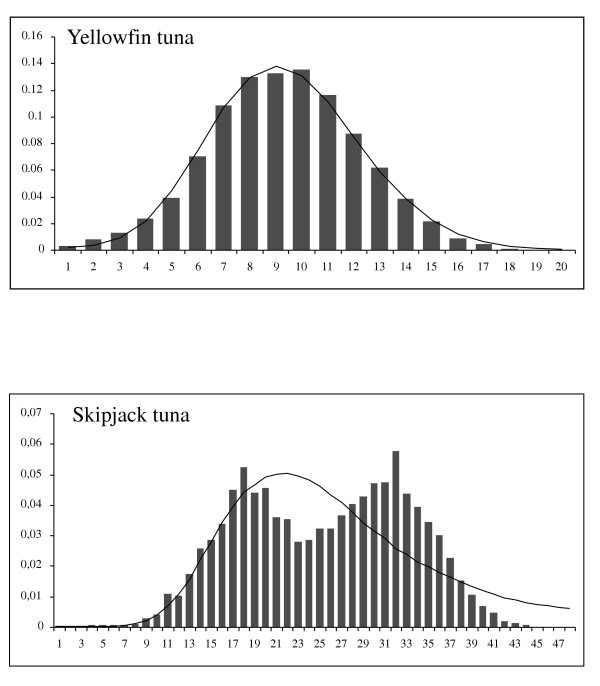
Mismatch distributions for the entire sample of a) yellowfin tuna and b) skipjack tuna. The solid bars in the histograms represent the observed pairwise differences between haplotypes and the curves the expected distribution under the sudden expansion model.

The unimodal mismatch distribution for the pooled sample of yellowfin tuna (Figure 2) and a significant Tajima *D *test (Table 1) both suggest the historical expansion of this population. In sharp contrast, the skipjack mismatch distribution is bimodal (Figure 2) and the neutrality test is not significant (Table 2), suggesting that the effective size of the skipjack population has been large and stable for a long period. In fact, the value of θ_0 _suggests that the number of females in the skipjack population was originally large (N_e0 _= 320,000) whereas the effective number of yellowfin tuna females prior to expansion was small (N_e0 _= 823). However, the values of θ_1 _suggest large long-term numbers of effective female breeders (N_e1_) of about 98 million for skipjack tuna and about 10 million for yellowfin tuna.

## Discussion

Compared to other scombroid species with cosmopolitan distributions, we found substantially less genetic differentiation between Atlantic and Pacific sub-populations of yellowfin tuna and no inter-oceanic genetic differentiation of skipjack tuna. For instance, in bigeye tuna, there are two highly divergent mtDNA clades, one is cosmopolitan and the other is endemic to the Atlantic [12,13]. This asymmetric distribution of Atlantic and Pacific clades has been observed in other large pelagic fishes as well, including swordfish [16,17], blue marlin [18], and sailfish [19,26]. Similarly, the striped marlin (Pacific) and white marlin (Atlantic) and the Atlantic bluefin tuna and the Pacific bluefin tuna are considered to be pairs of sister species [19-21,26]. Thus, significant differentiation has occurred between the Atlantic and Pacific populations of many large pelagic species. Why are skipjack and yellowfin tuna populations different? One possibility is that in contrast to other large pelagic species, sufficient gene flow occurs to prevent inter-oceanic genetic differentiation. However, this explanation is contrary to the distribution patterns of tunas and billfish [27,28]. For instance, bigeye tuna has a distribution of catches that would suggest a population continuum from the Indian Ocean to South Atlantic waters along the east and west coasts of Africa. However, bigeye samples show a marked inter-oceanic differentiation [12,13]. In the Indian Ocean, the presence of skipjack and yellowfin tuna south of 20° S, is confined to the warm waters associated with the Agulhas currents. Seasonal movements do occur around the Cape of Good Hope via the Agulhas current, but it appears that the migrant tuna and other pelagic species return to the Indian Ocean as the seasons change [13,29]. In fact, the distribution patterns of these two species in the South Atlantic along the African coast of are remarkably similar to both blue marlin and sailfish, two species that show a pronounced differentiation between Indo-Pacific and Atlantic populations. It should be noted, however, that the presence of "Pacific" mitochondrial DNA clades in Atlantic subpopulations of many tuna species indicates that inter-oceanic migration has occurred in the past. However, contemporary levels of inter-oceanic genetic differentiation for these species indicate that current levels of gene flow are absent or severely reduced. Thus, current distribution patterns of skipjack tuna and yellowfin tuna are not consistent with substantial levels of inter-oceanic gene flow. Certainly, there is no evidence to suggest that gene flow is occurring or has occurred at levels higher in these two species than in any of the other pelagic species where interoceanic differentiation has been demonstrated.

An alternative explanation to account for the lack of inter-oceanic differentiation in skipjack tuna and yellowfin tuna, is that the time since population expansion began has not been sufficient to allow for the populations to become differentiated. Assuming the very conservative mutation rate for CR-I of 4.9% per million years, a generation time 3.5 years, and the tau value 8.52, expansion of yellowfin tuna occurred about 522 Ky ago. By comparison, the estimated time for genetic differentiation of the Atlantic and Pacific populations of swordfish and of the Atlantic and Pacific bluefin tunas, using the same mutation rate, is very similar (450–470 Ky), and an even shorter time (170 Ky) is sufficient to explain the substantial genetic differentiation between the Atlantic and Mediterranean swordfish populations [30]. Thus, time since expansion cannot explain the absence of inter-oceanic differentiation in these species. Therefore, we propose that much larger effective population sizes are the primary factor responsible for the similarity of the Atlantic and Pacific sub-populations of skipjack and yellowfin tuna.

Among large pelagic species, the female effective population size can be estimated from the demographic estimates obtained from the CR-I sequences of Atlantic bluefin tuna and swordfish. Assuming a mutation rate of 4.9% per million years and a generation time of 6 years, the female N_e _estimates are 900,000 for bluefin tuna and 800,000 for swordfish [30]. Effective population sizes have not been estimated for other species of tunas, but annual harvest data are generally used as a proxy for abundance since the commercial harvest of large fish has become quite efficient throughout the world's oceans and harvest restrictions have only incremental impact on the total harvest of any species. Furthermore, haplotypic diversities of tuna mitochondrial DNAs are approximately 99% in all tuna species [12,31], suggesting that female reproductive variance is small. Accordingly, census population size can be expected to be proportional to effective population size in tuna species. The estimated number of skipjack and yellowfin caught in the year 2002 is 670 million and 130 million, respectively (Table 8). The catch of all other tunas was 24 million individuals or less. Thus, the abundance of skipjack tuna is approximately 5 times that of yellowfin, and more than 300 times that of Atlantic bluefin tuna and swordfish. These estimates of abundance from fisheries data appear to correspond well with the estimated female N_ef _values. The observed correspondence does not take into consideration dramatic changes in abundance of some of these species over the last 20 years, nor sex ratio differences, or the age distribution of the catch (e.g., number of mature females). However, the comparison supports the hypothesis that skipjack tuna and yellowfin tuna, which are the two most abundant species, also have the largest effective population sizes, and the lowest amounts of genetic partitioning compared to other scombroid fishes.

**Table 8:** Worldwide Tuna Catch Data for the Year 2002 [1]

Species	Catch (metric tons)	Ave. Wt. (kg)	Est. Number Fish Harvested*
skipjack	2.0 × 10^6^	3	670 × 10^6^
yellowfin	1.3 × 10^6^	5–20	130 × 10^6^
bigeye	0.43 × 10^6^	15–20	24 × 10^6^
albacore	0.24 × 10^6^	9–20	16 × 10^6^
longtail	0.125 × 10^6^	15–20	6.9 × 10^6^
Pacific bluefin	0.024 × 10^6^	7	3.4 × 10^6^
Atlantic bluefin	0.036 × 10^6^	17	2.1 × 10^6^

*Number of Atlantic bluefin tuna harvested obtained from ICCAT [51].

Bigeye tuna, and bluefin tuna have much greater levels of genetic differentiation between Atlantic and Pacific subpopulations when mitochondrial DNA control region sequences are compared [12,31]. However, their population sizes are 6 and 15 times lower than that of yellowfin and 30 and 75 times lower than that of skipjack, respectively. Furthermore, since bluefin tuna are a more temperate species, their effective population sizes may have been significantly lower than those observed in recent times whenever the northern hemisphere experienced glacial maxima. Thus, the relative differentiation of the Atlantic and Pacific subpopulations is consistent with the demographics of these species of tuna.

One exception to the patterns described above is albacore tuna. The distribution of albacore tuna mitochondrial DNAs does not appear to fit the patterns described above for the other temperate tuna species. Albacore abundance is similar to that of bigeye tuna and both species have two mtDNA clades. However, in albacore the two clades are not as well differentiated nor do they display the phylogeographic association observed for the bigeye tuna clades [12]. Instead, the bimodal mismatch distribution of pairwise differences in albacore mtDNA is concordant with very large long-term effective population sizes in contrast to the contemporary population size.

## Conclusion

Much lower levels of genetic differentiation were found among sub-populations of yellowfin tuna compared to those observed for other large tunas, probably due to the large population size of yellowfin tuna. Since skipjack tuna appear to have even larger population sizes than yellowfin tuna, it is not surprising that no genetic differentiation was observed between Atlantic and Pacific samples of these fish.

## Methods

Samples of yellowfin tuna were obtained from the eastern Pacific Ocean (near the equator at 110° W; n = 41), the Indian Ocean (n = 63), the Gulf of Mexico and the East Coast of Florida (n = 38) and the Gulf of Guinea (n = 100). Samples of skipjack tuna were obtained from the Northwest Atlantic (n = 31), off the coast of Brazil (n = 19), the eastern Pacific Ocean (n = 43), and the south Pacific near the Solomon Islands (n = 37). DNA isolation, mitochondrial DNA D-loop region amplification, and nucleotide sequence analyses have been described previously [12,32]. The number of segregating sites (S) was estimated with MEGA. Values of haplotypic diversity (h) [33], nucleotide diversity (π) [34] and the mean number of pairwise differences (K) were computed in ARLEQUIN ver. 2.0 [35]. Mitochondrial DNA haplotype phylogenies were estimated using neighbour-joining analyses [36] with Tamura-Nei distances (α = 0.5)) in MEGA [37]. The pair-deletion option was used when missing data, insertions, or deletions were present. Maximum-Parsimony (MP) [38,39] was carried using heuristic searches with the default options in PAUP* 4.0b10 [40]. Statistical support for the nodes was estimated with 1000 non-parametric bootstrap replicates [41]. All trees were rooted at midpoint. Analyses of molecular variance (AMOVA) [42] were performed to estimate the partitioning of genetic variation in regional hierarchical arrangements using ARLEQUIN.

The demographic history contained in the mtDNA CR-I sequence data was inferred using two approaches. First, the null hypothesis of neutrality may be rejected when a population has experienced population expansion [43]. Accordingly, Tajima's D test of neutrality [43,44] and its significance levels were estimated using DnaSP 4.00 [45] based on 1000 simulated re-samplings replicates. Alternatively, a population that has experienced a rapid expansion in the recent past shows smooth wave-like mismatch distribution [46,47]. Thus, mismatch distribution analyses, under the assumption of selective neutrality, were also used to evaluate possible historical events of population growth and decline [47,48]. Past demographic parameters, including τ [49], θ_0 _and θ_1 _and their probabilities [47] were estimated in ARLEQUIN taking into account the heterogeneity of mutation rates [35].

For the analysis of the mitochondrial cytochrome b gene, a 650 bp fragment of the skipjack tuna cytochrome b gene was amplified using primers CB3 (GGCAAATAGGAARTATCATTC) and GLUDG (TGACTTGAARAACCAYCGTTG) [50]. Alleles were identified by determining the nucleotide sequence of the amplified fragment. For yellowfin tuna, the ATCO gene region was amplified using primers H9342 (GCCATATCGTAGCCCTTTTTG) and L8562 (CTTCGACCAATTTATGAGCCC) [24]. The amplified fragments were digested with either *Dde*I or *Hpy*CH4III and the digestion products were resolved by electrophoresis in a 1.2% agarose gel.

## Authors' contributions

BE, JV, and JAB conceived the study, supervised the genetic studies, analyzed the data and wrote the manuscript. JAB, AL, and ET performed the DNA sequence analyses. BE, DB, ET, LL, and KC performed the DNA amplifications and RFLP analyses.

## References

[B1] FAO Yearbooks of fishery statistics summary tables. http://www.fao.org/fi/statist/statist.asp.

[B2] Scoles DR, Graves JE (1993). Genetic analysis of the population structure of yellowfin tuna, Thunnus albacares, from the Pacific Ocean. Fishery Bulletin.

[B3] Ward RD, Elliot NG, Innes BH, Smolenski AJ, Grewe PM (1997). Global population structure of yellowfin tuna, Thunnus albacares, inferred from allozyme and mitochondrial DNA variation. Fishery Bulletin.

[B4] Sharp GD, Sharp GD and Dizon AE (1978). Behavioral and physiological properties of tunas and their effects on vulnerability to fishing gear. The Physiological Ecology of Tunas.

[B5] Ward RD, Elliot NG, Grewe PM, Smolenski AJ (1994). Allozyme and mitochondrial DNA variation in yellowfin tuna (Thunnus albacares) from the Pacific Ocean. Marine Biology.

[B6] Schaefer KM (2001). Assessment of skipjack tuna (Katsuwanus pelamis) spawning activity in the eastern Pacific Ocean. Fishery Bulletin.

[B7] Fujino K (1976). Subpopulation identification of skipjack tuna specimens from the southwestern Pacific Ocean. Bulletin of the Japanese Society of Scientific Fisheries.

[B8] Richardson BJ (1983). Distribution of protein variation in skipjack tuna (Katsuwonus pelamis) from the central and south-western Pacific. Australian Journal of Marine and Freshwater Fisheries.

[B9] Fujino K (1996). Genetically distinct skipjack tuna subpopulations appeared in the central and western Pacific Ocean. Fisheries Science.

[B10] Fujino K (1981). Genetic diversity of skipjack tuna in the Atlantic, Indian, and Pacific Oceans. Bulletin of the Japanese Society of Scientific Fisheries.

[B11] Graves JE, Ferris SD, Dizon AE (1984). Close genetic similarity of Atlantic and Pacific skipjack tuna (Katsuwonus pelamis) demonstrated with restriction endonuclease analysis of mitochondrial DNA. Marine Biology.

[B12] Alvarado Bremer JR, Stequert B, Robertson NW, Ely B (1998). Genetic evidence for inter-oceanic subdivision of bigeye tuna (Thunnus obesus) populations. Marine Biology.

[B13] Chow S, Okamoto H, Miyabe N, Hiramatsu K, Barut N (2000). Genetic divergence between Atlantic and Indo-Pacific stocks of bigeye tuna (Thunnus obesus) and admixture around South Africa. Molecular Ecology.

[B14] Chow S, Ushiama H (1995). Global population structure of albacore (Thunnus alalunga) inferred by RFLP analysis of the mitochondrial ATPase gene. Marine Biology.

[B15] Viñas J, Alvarado Bremer JR, Pla C (2004). Inter-oceanic genetic differentiation among albacore (Thunnus alalunga) populations. Marine Biology.

[B16] Alvarado Bremer JR, Baker AJ, Mejuto J (1995). Mitochondrial DNA control region sequences indicate extensive mixing of swordfish (Xiphias gladius) populations in the Atlantic Ocean. Can J Fish Aquat Sci.

[B17] Rosel PE, Block BA (1996). Mitochondrial control region variability and global population structure in the swordfish, Xiphias gladius. Marine Biology.

[B18] Finnerty JR, Block BA (1992). Direct sequencing of mitochondrial DNA detects highly divergent haplotypes in blue marlin (Makaira nigricans). Molecular Marine Biology and Biotechnology.

[B19] Graves JE, McDowell JR (1995). Inter-ocean genetic-divergence of istiophorid billfishes. Marine Biology.

[B20] Collette BB, Reeb C, Block BA, Block BA and Stevens ED (2001). Systematics of the tunas and mackerels (Scombridae). Tuna: Physiology, Ecology, and Evolution, Fish Physiology Series.

[B21] Collette BB (1999). Mackerels, molecules, and morphology. Proc 5th Indo-Pacific Fish Conf, Nouméa, 1997.

[B22] Chow S, Kishino H (1995). Phylogenetic relationships between tuna species of the genus Thunnus (Scombridae: Teleostei): Inconsistent implications from morphology, nuclear and mitochondrial genomes. J Mol Evol.

[B23] Takeyama H, Chow S, Tsuzuki H, Matsunaga T (2001). Mitochondrial DNA sequence variation within and between tuna Thunnus species and its application to species identification. Journal of Fish Biology.

[B24] Chow S, Inoue S (1993). Intra- and interspecies restriction fragment length polymorphism in mitochondrial genes of Thunnus tuna species. Bulletin of the National Research Institute of Far Seas Fisheries.

[B25] Terol J, Mascarell R, Fernandez-Pedrosa V, Perez-Alonso M (2002). Statistical validation of the identification of tuna species: Bootstrap analysis of mitochondrial DNA sequences. J Agr Food Chem.

[B26] Graves JE, McDowell JR (2003). Stock structure of the world's istiophorid billfishes: a genetic perspective. Marine and Freshwater Research.

[B27] IOTC (2004). Report of the sixth session of the IOTC working party on tropical tunas.

[B28] ICCAT (2002). Report for the biennial period 2000-2001. Part II.

[B29] Talbot FH, Penrith MJ (1962). Tunnies and marlins of South Africa. Nature.

[B30] Alvarado Bremer JR, Viñas J, Mejuto J, Ely B, Pla C (2005). Comparative phylogeography of Atlantic bluefin tuna and swordfish: The combined effects of vicariance, secondary contact, introgression, and population expansion on the regional phylogenies of two highly migratory pelgaic fishes. Molecular Phylogenetics and Evolution.

[B31] Alvarado Bremer JR, Naseri I, Ely B (1997). Orthodox and unorthodox phylogenetic relationships among tunas revealed by the nucleotide sequence analysis of the mitochondrial DNA control region. Journal of Fish Biology.

[B32] Alvarado Bremer JR, Mejuto J, Greig TW, Ely B (1996). Global population structure of the swordfish (Xiphias gladius L) as revealed by analysis of the mitochondrial DNA control region. J Exp Mar Biol Ecol.

[B33] Nei M, Tajima F (1981). Genetic drift and estimation of effective population size. Genetics.

[B34] Nei M (1987). Molecular Evolutionary Genetics.

[B35] Schneider S, Roessli D, Excoffier L (2000). Arlequin: A software for population genetics data analysis.

[B36] Saitou N, Nei M (1987). The neighbor-joining method - a new method for reconstructing phylogenetic trees. Mol Biol Evol.

[B37] Kumar S, Tamura K, Jakobsen IB, Nei M (2001). MEGA2: molecular evolutionary genetics analysis software. Bioinformatics.

[B38] Kluge AG, Farris JS (1969). Quantitative phyletics and the evolution of anurans. Systematic Zoology.

[B39] Fitch WM (1971). Towards defining the course of evolution: minimal change for a specific tree topology. Systematic Zoology.

[B40] Swofford DL (2000). PAUP*. Phylogenetic analysis using parsimony (* and other methods).

[B41] Felsenstein J (1985). Confidence limits on phylogenies: an approach using the bootstrap. Evolution.

[B42] Excoffier L, Smouse PE, Quattro JM (1992). Analysis of molecular variance inferred from metric distances among DNA haplotypes: application to human mitochondrial DNA restriction data. Genetics.

[B43] Tajima F (1989). The effect of change in population size on DNA polymorphism. Genetics.

[B44] Tajima F (1989). Statistical method for testing the neutral mutation hypothesis by DNA polymorphism. Genetics.

[B45] Rozas J, Sachez-del Barrio JC, Messeguer X, Rozas R (2003). DnaSP, DNA polymorphism analyses by the coalescent and other methods. Bioinformatics.

[B46] Slatkin M, Hudson R (1991). Pairwise comparisons of mitochondrial DNA sequences in stable and exponentially growing populations. Genetics.

[B47] Rogers AR, Harpending H (1992). Population growth makes waves in the distribution of pairwise genetic differences. Molecular Biology and Evolution.

[B48] Rogers AR (1995). Genetic evidence for a Pleistocene explosion. Evolution.

[B49] Li WH (1977). Distribution of nucleotide differences between two randomly chosen cistrons in a finite population. Genetics.

[B50] Hillis DM, Moritz C, Mable BK (1996). Molecular Systematics.

[B51] ICCAT (2003). Report of the 2002 Atlantic bluefin tuna stock assessment session: ; Madrid..

